# CD72 downregulation on DN2 B cells is associated with disease activity and resistance to rituximab in systemic lupus erythematosus

**DOI:** 10.1093/rheumatology/keag097

**Published:** 2026-02-18

**Authors:** Kittikorn Wangriatisak, Wenqi Huang, Giorgio Sechi, Vilija Oke, Karine Chemin, Caroline Grönwall, Iva Gunnarsson, Vivianne Malmström, Francesca Faustini

**Affiliations:** Division of Rheumatology, Department of Medicine Solna, Karolinska Institutet, Karolinska University Hospital, Stockholm, Sweden; Center for Molecular Medicine, Karolinska Institutet, Stockholm, Sweden; Division of Rheumatology, Department of Medicine Solna, Karolinska Institutet, Karolinska University Hospital, Stockholm, Sweden; Center for Molecular Medicine, Karolinska Institutet, Stockholm, Sweden; Division of Rheumatology, Department of Medicine Solna, Karolinska Institutet, Karolinska University Hospital, Stockholm, Sweden; Center for Molecular Medicine, Karolinska Institutet, Stockholm, Sweden; Division of Rheumatology, Department of Medicine Solna, Karolinska Institutet, Karolinska University Hospital, Stockholm, Sweden; Centre for Rheumatology, Academic Specialist Centre, Stockholm Region, Sweden; Division of Rheumatology, Department of Medicine Solna, Karolinska Institutet, Karolinska University Hospital, Stockholm, Sweden; Center for Molecular Medicine, Karolinska Institutet, Stockholm, Sweden; Division of Rheumatology, Department of Medicine Solna, Karolinska Institutet, Karolinska University Hospital, Stockholm, Sweden; Center for Molecular Medicine, Karolinska Institutet, Stockholm, Sweden; Division of Rheumatology, Department of Medicine Solna, Karolinska Institutet, Karolinska University Hospital, Stockholm, Sweden; Medicine Unit Dermatology, Gastroenterology, Rheumatology; Section of Rheumatology, Karolinska University Hospital, Stockholm, Sweden; Division of Rheumatology, Department of Medicine Solna, Karolinska Institutet, Karolinska University Hospital, Stockholm, Sweden; Center for Molecular Medicine, Karolinska Institutet, Stockholm, Sweden; Division of Rheumatology, Department of Medicine Solna, Karolinska Institutet, Karolinska University Hospital, Stockholm, Sweden; Medicine Unit Dermatology, Gastroenterology, Rheumatology; Section of Rheumatology, Karolinska University Hospital, Stockholm, Sweden

**Keywords:** B cells, CD72, double-negative 2, systemic lupus erythematosus, rituximab

## Abstract

**Objectives:**

In SLE, expanded B cell subtypes like double-negative 2 (DN2) may harbour autoreactivity, which may be linked to impaired checkpoint regulation. We investigated checkpoint molecule CD72 on B cells, its clinical associations, and dynamic changes upon rituximab (RTX) treatment.

**Methods:**

Thirty SLE patients (26 with active disease) were studied. Seven received RTX treatment with sampling at baseline, 3 months and 6 months. B cell phenotypes were analysed using spectral flow cytometry, and clinical associations were evaluated. B cell receptor (BCR) signalling was studied through downstream phosphorylation *in vitro*.

**Results:**

Patients with active SLE showed increased frequencies of CD72-negative B cells in switched memory (SWM), activated naïve (aNAV), DN2 and plasmablast (PB) subsets compared with healthy controls (HCs). CD72-negative DN2 cells (CD27-IgD-CD21-CD11c+) were elevated in LN patients. These cells expressed higher CD95 and lower CD20 compared with HCs and canonical SLE DN2. Upon BCR stimulation, lupus CD72-negative SWM and DN2 B cells displayed increased pSYK phosphorylation compared with their CD72-positive counterparts and the overall cell population. CD72-negative DN2 frequencies associated positively with SLEDAI-2K and inversely with C3 levels and were increased in anti-dsDNA-positive patients. After RTX treatment, the remaining DN2 cells were primarily CD72-negative at 3 months.

**Conclusion:**

The DN2 B cell subset have been associated with autoimmunity. We showed that DN2 B cells in SLE have reduced CD72 expression, which is associated with anti-dsDNA positivity, complement consumption, and enhanced BCR downstream signalling. The relative resistance of CD72-negative B cells to RTX indicates that an impaired checkpoint programme in lupus B cells may potentially contribute to disease relapse. These findings require validation in larger cohorts.

## Graphical abstract

**Graphical Abstract:**
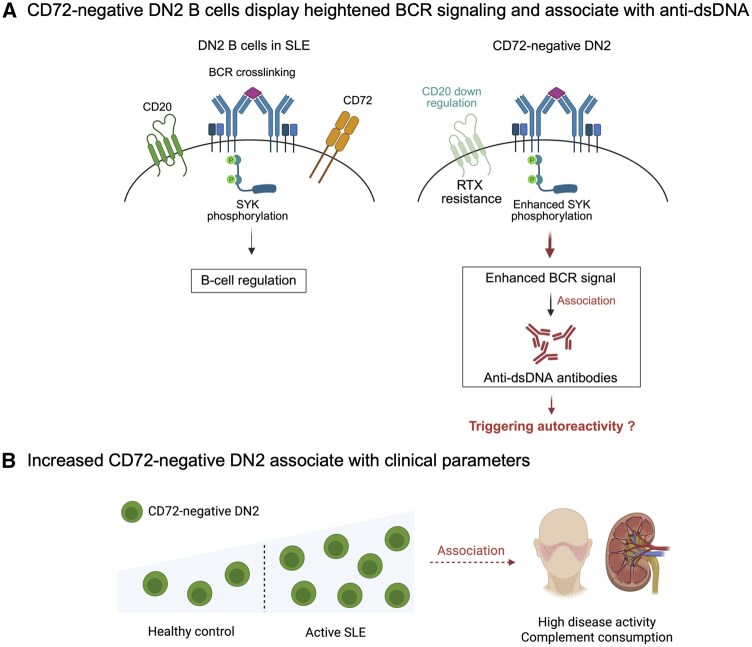

Rheumatology key messages

- Downregulation of CD72 on DN2 B cells is a feature of B-cell dysregulation in SLE
- CD72-negative DN2 B cells show heightened BCR signalling and are associated with anti-dsDNA positivity and low C3 levels
- Further decrease in CD72 expression on DN2 B cells post-treatment suggests persistent immune checkpoint impairment

## Introduction

The generation of pathogenic autoimmune B cell clones is a central event in autoimmune diseases [1, 2]. Breakdown of tolerance is a primary event that precedes overt clinical disease [3], in turn characterized by expansion of the antibody repertoire as highlighted by studies across various diseases [4]. This is especially true for SLE, a clinically protean disease characterized by widespread and profound disruption of B cell tolerance.

There is great diversity in circulating B cells, and the autoreactive clones in disease are part of this complex repertoire. Hence, studying autoreactive B cells presents challenges due to their low numbers and the difficulties in determining their antigen specificity [2]. Moreover, the autoreactive B cells are hypothesized to be driven by different mechanisms, undergoing activation and proliferation both via B cell receptor signalling and via Toll-like receptor (TLR) engagement [5]. Immune responses are characterized by recognition (of a foreign antigen, e.g.), followed by activation, expansion and wearing-off of the response, by intrinsic cellular pathways (checkpoint molecules), or by external intervention (e.g. regulatory lymphocytes). Autoimmune conditions are often characterized by reiterated antigen stimulation (i.e. repeated or chronic stimulation) of the B cells, which may be associated with altered expression of checkpoint molecules, i.e. cell surface molecules whose canonical function is linked to switching off (negatively regulating) B cell activation and maintaining immune tolerance, or returning to homeostatic balance (e.g. when an infection is controlled). The condition of repeated stimulation (by autoantigens) contributes to impairing the capacity of the immune system to return to homeostatic balance.

CD72, a type II lectin transmembrane protein, serves as a negative regulator of B cell receptor (BCR) signalling. This molecule contains an immunoreceptor tyrosine-based inhibition motif (ITIM), which can recruit SHP-1 (Src homology region 2 domain-containing phosphatase-1) to attenuate BCR signalling [6]. Through this inhibitory pathway, CD72 may contribute to the regulation of B cell activation thresholds and immune tolerance. In the context of SLE, the CD72 molecule has been mechanistically linked to tolerance towards nuclear antigens (Sm/RNP and ribosomes) implicated in the sensing of nucleic acids [6, 7]. Nevertheless, data on the spectrum of expression and its functional role across different peripheral B cell subsets remain scarce. Notably, previous studies have identified double-negative 2 cells (DN2, CD27-IgD-CD21-CD11c+) as a subset particularly associated with active disease in SLE [8].

In this study we aimed to investigate the expression of CD72 in human SLE-derived B cells, exploring its clinical associations and possible dynamics upon treatment.

## Methods

### Patients and samples

Patients were older than 18 years and met the 1982 ACR and 2012 SLICC classification criteria for SLE [9, 10]. Patients received standard of care treatment. Collected data consisted of demographics, clinical manifestations, and laboratory parameters. Exclusion criteria were presence of overlapping syndromes, and ongoing or suspected cancer or infection.

Blood samples from 30 SLE patients were included (inactive: SLEDAI-2K score < 4, *n* = 4 and active: SLEDAI-2K score ≥ 4, *n* = 26). The patients selected as inactive were in principle allowed to be in a condition of serologically active and clinically quiescent disease. Among the active patients, seven received rituximab (RTX) treatment and were sampled longitudinally at baseline, 3 months and 6 months. All samples were analysed for B cell phenotyping (Supplementary Table S1), and samples from four active SLE patients were included in the intracellular BCR phosphoflow experiments (Supplementary Table S2). Samples from age- and sex-matched healthy control blood donors (HCs, *n* = 13) were included.

### Detection of CD72-expressing B-cells by full-spectrum flow cytometry

Peripheral blood mononuclear cells (PBMCs) were isolated by Ficoll-Paque (Cytiva) separation and stained with fluorochrome-labelled antibodies (Supplementary Table S3). Cells were co-stained with fixable viability dye eFluor 506 (1:1000, Invitrogen). Data were acquired using the Cytek Aurora spectral flow cytometer (5-laser, 355 nm, 405 nm, 488 nm, 561 nm and 640 nm). Both supervised and unsupervised analyses were performed.

### Intracellular phosphoflow staining

PBMCs (1 × 10^6^ cells) were incubated with fixable viability dye eFluor 506 for 10 min at 4°C. Thereafter, cells were washed twice with PBS and surface-stained with antibodies (Supplementary Table S4) for 20 min at 4°C. Cells were subsequently washed and rested in 2% fetal bovine serum (FBS)/RPMI (2% FBS, 2 mM L-glutamine, 100 U/ml penicillin, 100 µg/ml streptomycin and 10 mM HEPES) at 37°C for 1 hour. Then, cells were stimulated with 20 µg/ml of F(ab)2 anti-IgM and anti-IgG (Jackson ImmunoResearch) at 37°C for 2 and 5 min to detect pSYK and pERK, respectively. Cells were fixed, permeabilized and stained with antibodies specific for phosphorylated SYK (pY348) and ERK (pY204) followed by analysis using a 4-laser Cytek Aurora spectral flow cytometer.

### Statistical analysis

Data were analysed using GraphPad Prism 9.5.0 software and are represented as medians (Q1–Q3). Medians were compared using the Mann–Whitney *U* test (two groups) or Kruskal–Wallis (>2 groups) with Dunn’s correction for the multiple comparison test. Spearman’s test was applied to investigate correlations. *P*-values of <0.05 were considered statistically significant.

## Results

### CD72 expression is decreased in SLE across B cell subsets, most prominently in DN2

**Figure 1:**
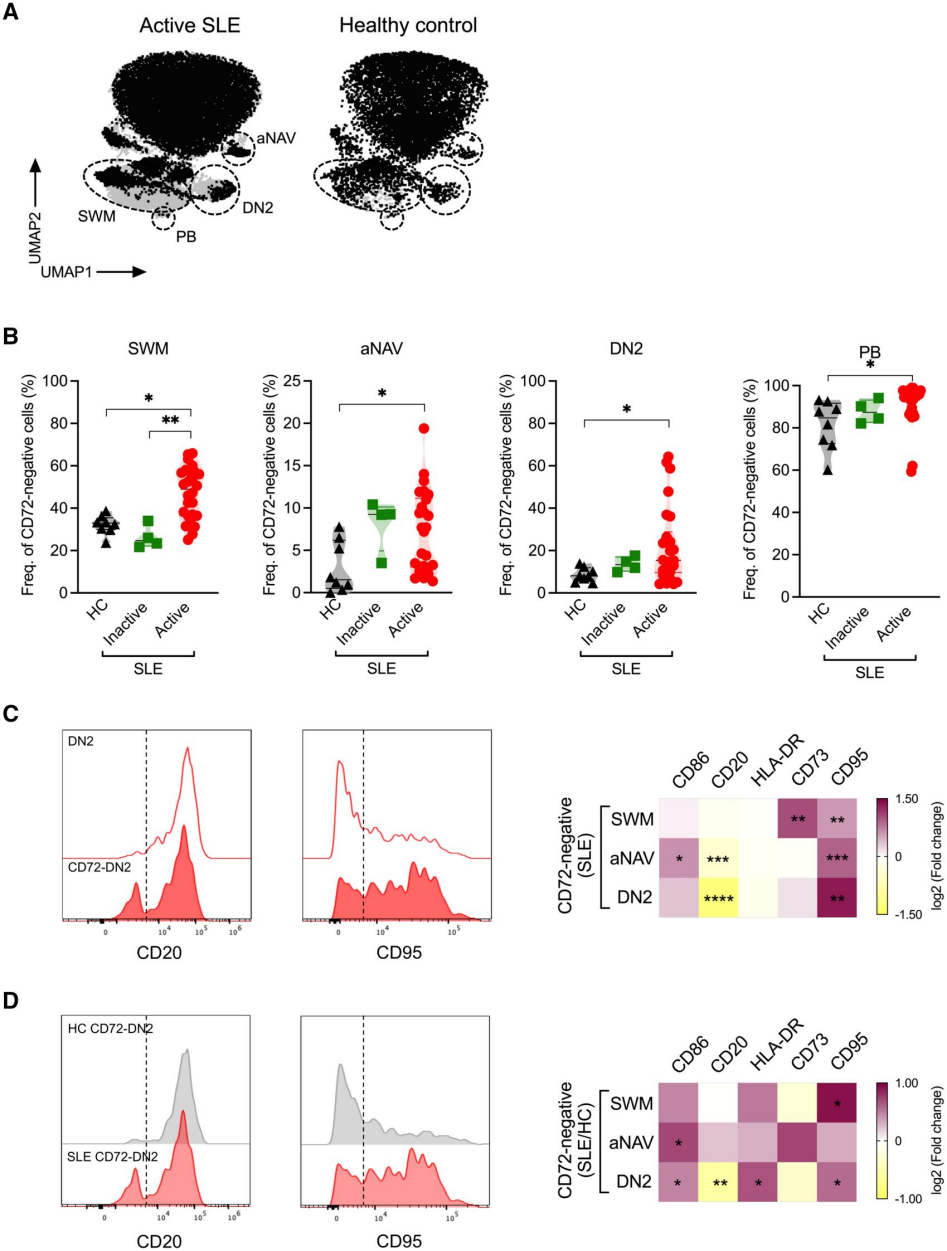
Expression of surface CD72 in various B cell subsets. (A) Uniform manifold approximation and projections (UMAPs) showing CD72 expression on CD19+B cells (50 000 events) from one active SLE patient and a healthy control (HC). (B) Frequency of CD72-negative cells in switched memory (SWM), activated naïve (aNAV), double-negative 2 (DN2) and plasmablast (PB) subsets from patients with active SLE (*n* = 26), patients with inactive SLE (*n* = 4) and HCs (*n* = 8). (C, D) Representative histogram overlays of CD20 and CD95 expression (left) and a heat map showing fold changes in median fluorescence intensity (MFI) of marker expression (right) from (C) CD72-negative cells relative to overall cells in each subset (*n* = 26) and (D) CD72-negative cells in patients with SLE relative to HCs (*n* = 8). Dashed lines depict the cut-off between positive and negative populations. Data in (B) are represented as median and interquartile range and were analysed using the Kruskal–Wallis test; *P* values were corrected using Dunn’s test for multiple comparisons. Data in (C, D) were analysed using the Mann–Whitney *U* test. Only statistically significant *P* values of <0.05 are presented. *p < 0.05; **p < 0.01; ***p < 0.001; ****p < 0.0001

We first investigated how CD72 was expressed on major B cell subsets in SLE patients and HCs. While CD72 expression was superimposable on naïve phenotypes in patients and controls, we observed that switched memory (SWM; CD27^+^IgD^−^), double-negative 2 (DN2; CD27^−^IgD^−^CD21^−^CD11c^+^) and plasmablasts (PB; CD27^hi^CD38^hi^) exhibited reduced CD72 expression in patients compared with controls (Fig. 1A and Supplementary Fig. S1A and B).

We next focused on characterizing the proportions of CD72-negative cells within each defined B cell subset and their relation to disease activity and phenotype (Fig. 1B and Supplementary Fig. S1C). In patients, compared with HCs, the proportions of CD72-negative cells were expanded within SWM, activated naïve (aNAV), DN2 and PB phases. Moreover, the proportion of CD72-negative DN2 cells showed a significant positive correlation with that of CD72-negative aNAV phase (Supplementary Fig. S2).

We further proceeded to phenotypically characterize B cells lacking surface expression of CD72 with the expression of other surface markers. All CD72-negative B cell subpopulations within SWM, aNAV and DN2 B cell subsets, showed increased expression of CD95, while upregulation of activation markers, CD73 and CD86, was observed only in SWM and aNAV B cells (Fig. 1C). Notably, reduced CD20 expression was found in CD72-negative aNAV and DN2, with a more pronounced reduction in the DN2 subset (Fig. 1C). Similarly, differences were observed when comparing patients and HCs, with CD72-negative DN2 from patients displaying markedly lower CD20 expression, together with increased activation markers (CD86 and HLA-DR) and CD95, compared with their counterparts in the HCs (Fig. 1D).

### Loss of CD72 impacts BCR signalling more distinctly in SLE

**Figure 2:**
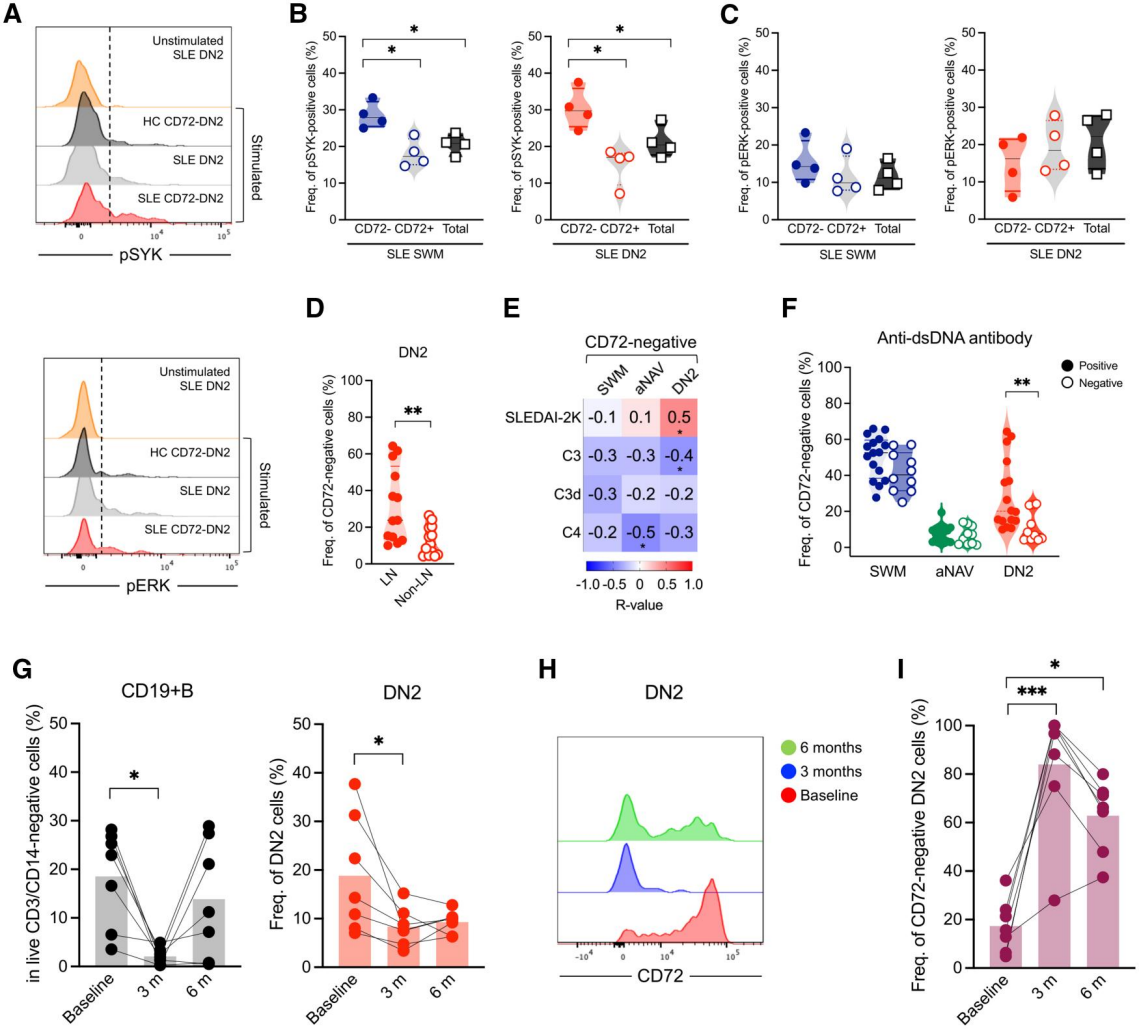
CD72-negative double-negative 2 (DN2) B cells are associated with anti-dsDNA positivity, complement consumption, and resistance to rituximab (RTX) treatment. (A) Representative histogram overlays of pSYK and pERK in stimulated cells [CD72-negative DN2 cells and total DN2 cells of patients and healthy controls (HCs)] compared with unstimulated SLE DN2 cells. Dashed lines depict the cut-off between positive and negative populations. (B) Frequency of pSYK-positive and (C) pERK-positive cells in CD72-negative switched memory (SWM) and DN2 cells compared with CD72-positive compartments and total SWM and DN2 cells in SLE patients (*n* = 4). (D) Frequency of CD72-negative cells in DN2 cells from LN (*n* = 13) and non-LN (*n* = 13) groups. (E) Correlation matrix heatmap of CD72-negative SWM, activated naïve (aNAV) and DN2 cell frequencies and clinical parameters in SLE patients (*n* = 26). The strength of the correlation is represented by varying intensity levels (−1 to 1). (F) Frequencies of CD72-negative SWM, aNAV and DN2 subsets cells in patients positive (*n* = 16) or negative (*n* = 10) for anti-dsDNA IgG. (G) Frequency of CD19+B cells and DN2 cells at baseline and follow-up time points (3 and 6 months) in patients (*n* = 7). (H) Histogram overlays showing CD72 expression on lupus DN2 cells at different time points. (I) Frequency of CD72-negative DN2 cells at baseline and after RTX treatment (3 and 6 months; *n* = 7). Data in (B–C and G–I) were analysed using the Kruskal–Wallis test, and *P* values were corrected using Dunn’s test for multiple comparisons. Data in (B and F) were analysed using the Mann–Whitney *U* test. Correlation analysis (in C) was performed using Spearman’s Rank coefficient (*r*). Only statistically significant *P* values of <0.05 are presented. *p < 0.05; **p < 0.01; ***p < 0.001.

To better understand the functional implications of the absent CD72 expression, we sought to determine the activation kinetics of the CD72-negative B cells, studying the expression of pSYK and pERK following stimulation (Fig. 2A). Loss of CD72 had a significant impact on BCR activation. In SLE, the CD72-negative cells within the total SWM and DN2 cells, showed increased proportions of pSYK positivity compared with their CD72-positive compartment and overall cell population (Fig. 2B), indicating a higher activation status. Similarly, overall CD72-negative cells in patients displayed increased SYK phosphorylation compared with HCs (Supplementary Fig. S3). In contrast, pERK proportions did not differ between patients and controls (Fig. 2C).

### CD72-negative B cells are associated with disease activity and clinical markers

We next looked at clinical associations. Importantly, CD72-negative cells were even more increased in patients with active disease. The patients with LN showed higher proportions of CD72-negative B cells, especially within the DN2 subset, in comparison with patients with other disease manifestations (non-LN) (Fig. 2D). CD72-negative DN2 cells of lupus patients were positively associated with the SLEDAI-2K score and inversely associated with C3 levels, a finding indicating that increase in these cells goes along with clinical and immunological activity (Fig. 2E). Additionally, CD72-negative aNAV cells were inversely associated with C4 levels. Interestingly, CD72-negative DN2 B cell frequencies were higher among patients with positive anti-dsDNA antibodies (Fig. 2F).

### CD72-negative DN2 cells are more resistant to B cell depletion

Seven of the patients recruited into the study underwent treatment with RTX after baseline sampling and had follow-up samples captured. Given the lower CD20 expression on CD72-negative DN2 cells, we examined how these cells behave following anti-CD20 B cell depleting treatment with the drug.

DN2 cells are known to express CD20 and, in line with this, we observed a decline in both CD19+ B cells and DN2 proportions in the short follow-up (Fig. 2G). When examining the fluorescence intensity of CD72 from baseline through 6 months (before to after treatment), we observed a lower intensity on the residual DN2 cells, with a concomitant increase in the proportion of CD72-negative cells within the DN2 subset. These findings may indicate a resistance of CD72- DN2 cells to anti-CD20 targeting (Fig. 2H and I).

## Discussion

In this work, we identified a potentially autoreactive B cell subset lacking expression of the checkpoint molecule CD72. As a first observation, we noted that, in patients with SLE, post-activated B cell phenotypes (SWM, DN2 and PB) showed lower proportions of CD72 expression compared with controls. In recent years, DN2 and PB have been characterized as predominant disease-associated subsets in SLE. These phenotypes are deemed to be central in extrafollicular reactions, i.e. B cell evolutionary pathways from which autoimmune clones are likely to emerge [8, 11]. The clustering of CD72-negative staining within these phenotypes of B cells is consistent with a lack of checkpoint control, which thereby could contribute to autoantibody generation. Our results are in line with the findings of previous studies that have also suggested that CD72 reduced expression plays a role in SLE development both in murine models and in human disease [6, 12, 13].

Notably, we found a predominant expression of the activation markers CD86 and HLA-DR as well as of CD95 in the CD72-negative DN2 cells, compared with the overall DN2 pool in patients and HCs, indicating an activated cell state that is associated with active disease [11, 14]. Moreover, the downregulation of CD20 on CD72-negative DN2 cells, which is consistent with B cell activation processes, has potential therapeutic implications [15]. In fact, with CD20 being the target of RTX, this observation calls into question which potentially pathogenic B cell phenotypes are susceptible to RTX action (and to that of other CD20-targeting drugs). To explore this, our longitudinal analyses in RTX-treated SLE patients showed retention of CD72 downregulation on remaining DN2 cells, despite an overall decline in DN2 frequencies similar to what we have previously observed [16]. These data suggest that CD72-negative DN2 cells may escape or be more refractory to depletion, potentially contributing to relapse or persistent disease activity.

Functionally, CD72-negative memory B cells (SWM and DN2) from patients showed a higher level of phosphorylation of the downstream SYK molecule, which is pivotal in BCR signalling events, after BCR stimulation. These findings indicate that the CD72-negative phenotype is more prone to persistent activation. Elevated expression of pSYK, both in basal conditions and upon BCR stimulation, has been previously described in SLE patients and appears as a feature of certain memory B cell subsets [17]. The observation of reduced CD72 expression in this context is in line with its described role as a negative regulator of BCR signalling, where its absence may enhance downstream signalling cascades [6, 12]. No difference in pERK levels between CD72-negative and -positive cells suggests that enhanced proximal signalling alone may be insufficient to activate the ERK/MAPK pathway, implying that pERK induction may require additional signals such as CD40-CD40L, TLRs or cytokine-receptors [18, 19]. A possible alternative explanation for the increased pSYK levels in CD72-negative SWM and DN2 B cells in SLE could be associated with BCR-hyporesponsiveness of these cells, whose activation, as previously shown, mainly takes place through TLR engagement [8, 20]. The understanding of this underlying mechanism is minimal and requires further investigation.

Regarding a possible role of CD72 loss in LN development [12], our analysis revealed an increased proportion of CD72-negative cells within the DN2 subset of patients with active LN. In addition, the presence of CD72-negative DN2 was associated with anti-dsDNA IgG antibody positivity and complement consumption in SLE patients. This might indicate that loss of CD72 expression on pathogenic B cells is associated with anti-dsDNA production and contributes to disease progression. However, the small sample size of LN patients did not allow stratification by histological subtype, and larger, well-characterized cohorts will be required in order to address this question.

In conclusion, we present further evidence of CD72 dysregulation in SLE B cells, with reduced expression in subsets related to extrafollicular responses, particularly DN2. Low CD72 is associated with anti-dsDNA positivity and complement consumption, indicating that loss of CD72 on DN2 may contribute to disease mechanisms. The persistent loss of CD72 on DN2 cells after treatment suggests that residual DN2 may impair the immune checkpoint programme and contribute to ongoing lupus activity.

The study was approved by the local Ethical Review Board, Regionala etikprövningsnämnden i Stockholm (2012/1550–31/3 with amendment 2019-02976 and 2001/152 with amendment 2014/39432). Patients gave written informed consent to participate. The study was conducted in accordance with the Declaration of Helsinki.

## Supplementary Material

keag097_Supplementary_Data

## Data Availability

The data underlying this article will be shared on reasonable request to the corresponding author.
