# Supplementary material for: CD72 downregulation on DN2 B cells is associated with disease activity and resistance to rituximab in systemic lupus erythematosus
Source: Rheumatology (Oxford). 2026 Feb 18;65(3):keag097. doi: 10.1093/rheumatology/keag097 (PMC13017721; doi:10.1093/rheumatology/keag097)
Supplement: keag097_Supplementary_Data [file keag097_supplementary_data.docx]

**Supplementary Table S1** Characteristics of systemic lupus erythematosus patients and healthy controls

| Categories | Features | SLE | | HC |
| --- | --- | --- | --- | --- |
|  |  | **Active** | **Inactive** |  |
| Demographic | No. of patients, No (%) | 26/30 (86%) | 4/30 (14%) | 8 |
|  | Age, years (#) | 39 (31-48) | 34.5 (31-46.5) | 38.5 (33.5-50.5) |
|  | Gender |  |  |  |
|  | - Male, No (%) - Female, No (%) | 2/26 (8%)  24/26 (92%) | 1/4 (25%)  3/4 (75%) | 1/7 (14%)  7/8 (86%) |
|  | No. of lupus nephritis, No (%) | 13/26 (50%) | 0/4 (0%) | ND |
|  | SLEDAI-2K score | 10 (6-13) | 0.5 (0-2) | ND |
|  | Disease duration, years (#) | 9 (4-17) | 2 (1.5-9.5) | ND |
| Laboratory parameters | Plasma creatinine, µmol/L (#) | 63 (50.5-77) | 76.5 (71-83.5) | ND |
|  | ESR rates, mm/h (#) | 34 (15-64) | 14.5 (10.5-24) | ND |
|  | C3 levels, g/L (#) | 0.7 (0.6-0.9) | 1.04 (0.87-1.13) | ND |
|  | C3d levels, g/L (#) | 8 (5.5-8) | 9 (4-22) | ND |
|  | C4 levels, g/L (#) | 0.1 (0.07-0.15) | 0.19 (0.15-0.32) | ND |
|  | Anti-dsDNA positive, No (#) | 16/26 (62%) | 1/4 (25%) | ND |
| Treatment at inclusion | Mean dose (Prednisolone equivalent, mg) | 11.25 | 3.12 | ND |
|  | Antimalarial | 17/26 (65%) | 3/4 (75%) | ND |
| *sDMARDs* | Azathioprine | 3/26 (11%) | 1/4 (25%) | ND |
|  | Mycophenolate mofetil | 3/26 (11%) | 1/4 (25%) | ND |
|  | Calcineurin inhibitors | 1/26 (4%) | 0/4 (0%) | ND |
| *bDMARDs* | Belimumab | 0/26 (0%) | 0/4 (0%) | ND |
|  | Rituximab | 7/26 (27%) | 1/4 (25%) | ND |

ND: Not done; SLE: Systemic Lupus Erythematosus; SLEDAI-2K: Systemic Lupus Erythematosus Disease Activity 2000; ESR: Erythrocyte Sedimentation Rate; C1q: Complement 1q; C3: Complement 3; C3d: Complement 3d; C4: Complement 4; Anti-dsDNA: Anti-double strand DNA; sDMARDs: synthetic disease-modifying antirheumatic drugs; bDMARDs: biologic disease-modifying antirheumatic drugs; # Median (Q1-Q3).

**Supplementary Table S2** Characteristics of SLE patients and healthy controls used in BCR phosphoflow study

| Categories | Features | SLE | HC | |
| --- | --- | --- | --- | --- |
| Demographic | No. of patients, No (%) | 4 | 5 | |
|  | Age, years (#) | 36.5 (32-47.5) | 41 (35-50.5) | |
|  | Gender |  |  | |
|  | - Male, No (%) - Female, No (%) | 0/4 (20%)  4/4 (100%) | 1/5 (20%)  4/5 (80%) | |
|  | SLEDAI-2K score | 11 (8-13) | ND | |
|  | Disease duration, years (#) | 20 (13.5-23) | ND | |
| Laboratory parameters | Plasma creatinine, µmol/L (#) | 59.5 (48.5-78.5) | ND | |
|  | ESR rates, mm/h (#) | 13.5 (10-15) | ND | |
|  | C3 levels, g/L (#) | 0.86 (0.68-0.89) | ND | |
|  | C4 levels, g/L (#) | 0.12 (0.08-0.25) | ND |  |
|  | Anti-dsDNA positive, N0 (#) | 1/4 (25%) | ND | |
| Treatment at inclusion | Mean dose (Prednisolone equivalent, mg) | 7.5 | ND | |
|  | Antimalarial | 3/4 (75%) | ND | |
| *sDMARDs* | Azathioprine | 1/4 (25%) | ND | |
|  | Mycophenolate mofetil | 0/4 (0%) | ND | |
|  | Calcineurin inhibitors | 0/4 (0%) | ND | |
| *bDMARDs* | Belimumab | 0/10 (0%) | ND | |
|  | Rituximab | 1/4 (25%) | ND | |

ND: Not done; SLE: Systemic Lupus Erythematosus; SLEDAI-2K: Systemic Lupus Erythematosus Disease Activity 2000; ESR: Erythrocyte Sedimentation Rate; C1q: Complement 1q; C3: Complement 3; C4: Complement 4; Anti-dsDNA: Anti-double strand DNA; sDMARDs: synthetic disease-modifying antirheumatic drugs; bDMARDs: biologic disease-modifying antirheumatic drugs; # Median (Q1-Q3). Patient received RTX after blood collection.

**Supplementary Table S3** Flow cytometry panel used for this study

| Antibody | Fluorophore | Clone | Company | Dilution | Catalogue No. |
| --- | --- | --- | --- | --- | --- |
| Fixable viability dye | Eflour506 | - | ThermoFisher | 1:1000 | 65-0866-14 |
| CD3 | Eflour506 | UCHT1 | Invitrogen | 1:25 | 69-0038-42 |
| CD14 | Eflour506 | 61D3 | Invitrogen | 1:25 | 69-0149-42 |
| IgD | BV480 | IA6-2 | BD | 1:200 | 566138 |
| CD20 | BUV395 | 2H7 | BD | 1:50 | 563782 |
| CD27 | APC/Fire810 | QA17A18 | Biolegend | 1:100 | 393214 |
| CD38 | BUV563 | HB7 | BD | 1:400 | 741446 |
| CD21 | BUV805 | B-ly4 | BD | 1:400 | 742008 |
| CD24 | BUV496 | ML5 | BD | 1:25 | 741143 |
| IgM | SB436 | SA-DA4 | Invitrogen | 1:50 | 62-9998-42 |
| IgA | VioBlue | IS11-8E10 | Miltenyi | 1:400 | 130-113-479 |
| IgG | BV421 | G18-145 | BD | 1:50 | 562581 |
| CD19 | BV570 | HIB19 | Biolegend | 1:25 | 302236 |
| CD45RB | BUV615 | MT4 (6B6) | BD | 1:800 | 751482 |
| CD138 | BV711 | MI15 | Biolegend | 1:25 | 356522 |
| CXCR5 | BV750 | J24D4 | Biolegend | 1:50 | 356942 |
| CD73 | BV785 | AD2 | Biolegend | 1:100 | 344028 |
| CD95 | PE/Cy5 | DX2 | Biolegend | 1:800 | 305610 |
| HLA-DR | BV650 | L243 | Biolegend | 1:400 | 307650 |
| CD11c | APC/Fire750 | S-HCL-3 | Biolegend | 1:400 | 371509 |
| CCR7 | PE/Fire810 | G043H7 | Biolegend | 1:100 | 353269 |
| CD72 | RB545 | J4-117 | BD | 1:200 | 756280 |
| BTLA | AF647 | MIH26 | Biolegend | 1:50 | 344519 |
| CD360 | PE | 17A12 | Biolegend | 1:50 | 359506 |
| SLAMF7 | BUV737 | 235614 | BD | 1:25 | 750833 |
| CD86 | BB515 | 2331 (FUN-1) | BD | 1:50 | 564544 |

**Supplementary Table S4** Flow cytometry panel used for BCR phosphowflow study

| Antibody | Fluorophore | Clone | Company | Dilution | Catalogue No. |
| --- | --- | --- | --- | --- | --- |
| Fixable viability dye | Eflour506 | - | ThermoFisher | 1:1000 | 65-0866-14 |
| CD3 | Eflour506 | UCHT1 | Invitrogen | 1:25 | 69-0038-42 |
| CD14 | Eflour506 | 61D3 | Invitrogen | 1:25 | 69-0149-42 |
| CD20 | PerCP | 2H7 | Biolegend | 1:20 | 302323 |
| CD27 | APC/Fire810 | QA17A18 | Biolegend | 1:100 | 393214 |
| CD38 | BV711 | HB7 | BD | 1:100 | 740830 |
| CD21 | FITC | B-ly4 | BD | 1:50 | 561372 |
| CD19 | BV570 | HIB19 | Biolegend | 1:25 | 302236 |
| IgD | BV480 | IA6-2 | BD | 1:200 | 566138 |
| CD72 | RB545 | J4-117 | BD | 1:200 | 756280 |
| CD11c | APC/Fire750 | S-HCL-3 | Biolegend | 1:400 | 371509 |
| CD95 | PE/Cy5 | DX2 | Biolegend | 1:800 | 305610 |
| pSYK (pY348) | PE | I120-722 | BD | 1:25 | 558529 |
| pERK (pY204) | AF647 | 20A | BD | 1:35 | 612593 |

**Supplementary Figure S1** Phenotype of B cells in the blood of SLE patients (**A**) Representative gating strategies of CD19+B-cells from one SLE patient. The five independent subpopulations were identified: plasmablast (PB; CD19^+^CD27^hi^CD38^hi^), switched memory (SWM; CD19^+^CD27^+^IgD^-^), unswitched memory (USW; CD19^+^CD27^+^IgD^+^), double negative (DN; CD19^+^CD27^-^IgD^-^) and naïve B-cells (NAV; CD19^+^CD27^-^IgD^+^). The subsets of DN and NAV B-cells were classified and grouped as double negative 1 (DN1; CD19^+^CD27^-^IgD^-^CD21^+^CD11c^-^), double negative 2 (DN2; CD19^+^CD27^-^IgD^-^CD21^-^CD11c^+^), double negative 3 (DN3; CD19^+^CD27^-^IgD^-^CD21^-^CD11c^-^), resting naïve (rNAV; CD19^+^CD27^-^IgD^+^CD21^+^CD11c^-^) and activated naïve (aNAV; CD19^+^CD27^-^IgD^+^CD21^-^CD11c^+^). (**B**) Flow cytometric plots represent CD72 expression among different B-cell subsets. (**C**) Frequency of CD72-negative cells in different B-cell subsets among patients with active (n = 26), inactive (n = 4) disease and HC (n = 8). Comparison of cell frequencies among different groups was analysed by Kruskal-Wallis test and *p* values were corrected by Dunn’s test for multiple comparisons.


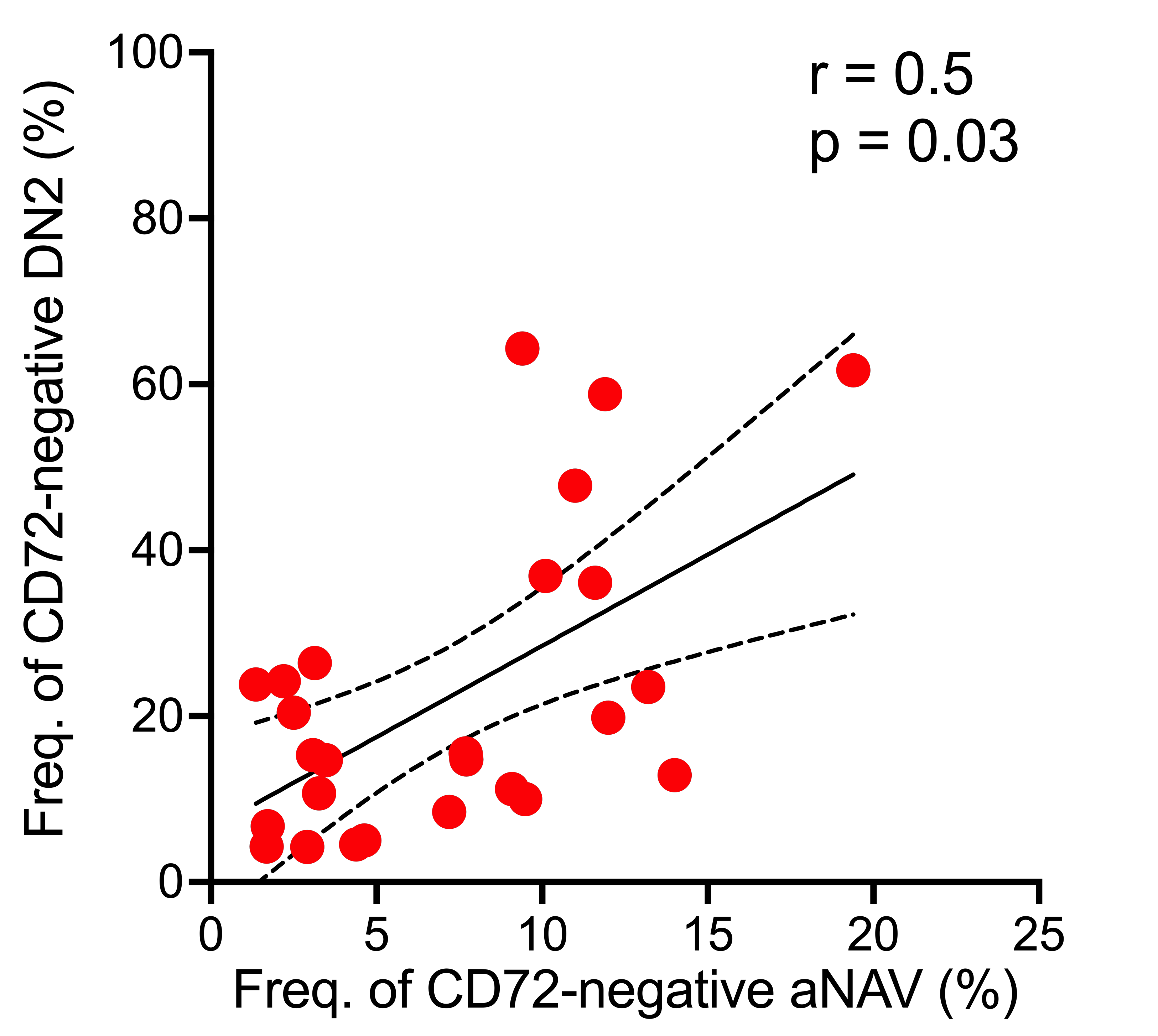


**Supplementary Figure S2** Correlation analysis between frequencies of CD72-negative DN2 with aNAV in active SLE patients (n = 26). Correlation analysis was done using Spearman’s Rank coefficient (*r*). Only statistically significant *p* values < 0.05 are presented.


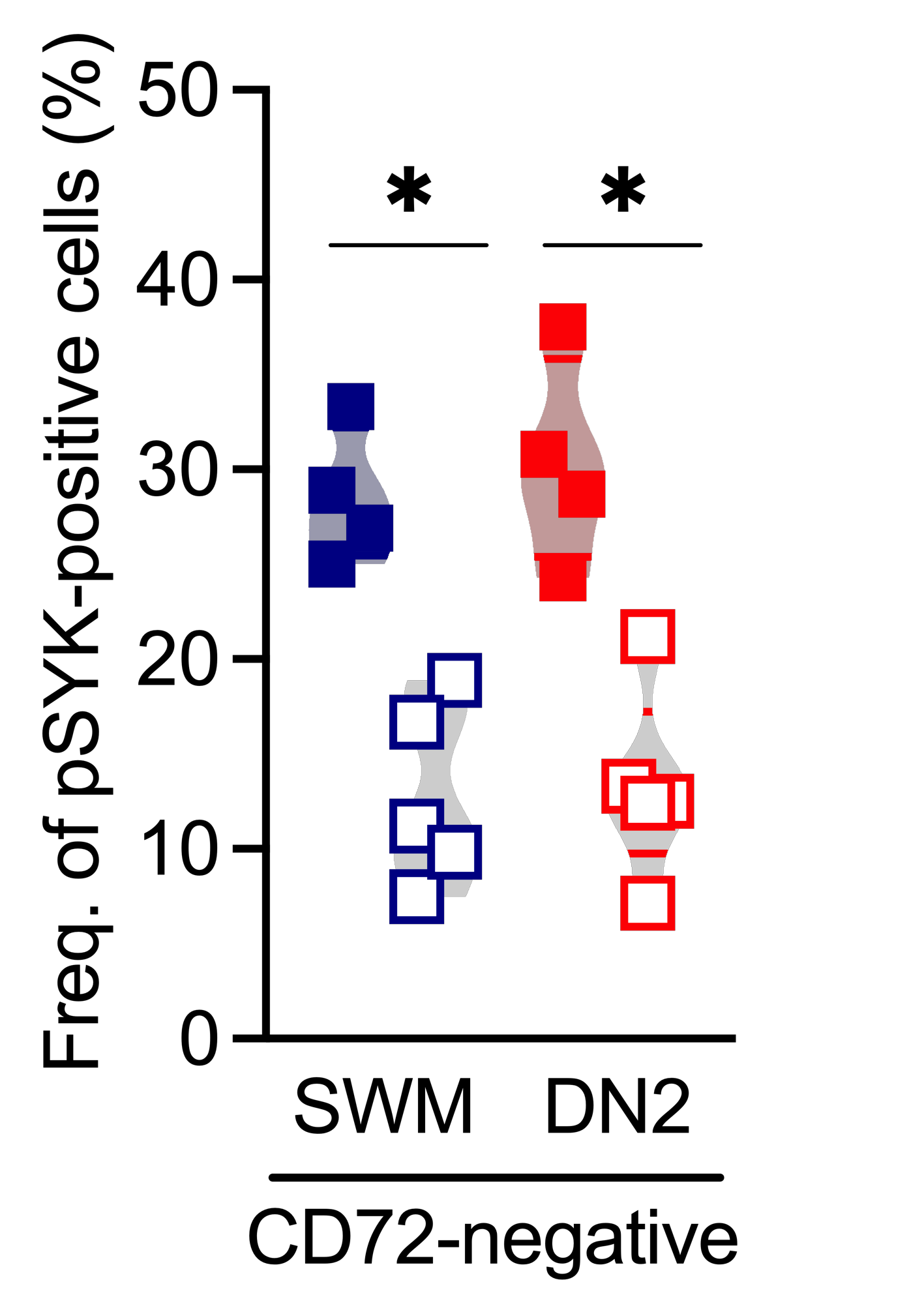

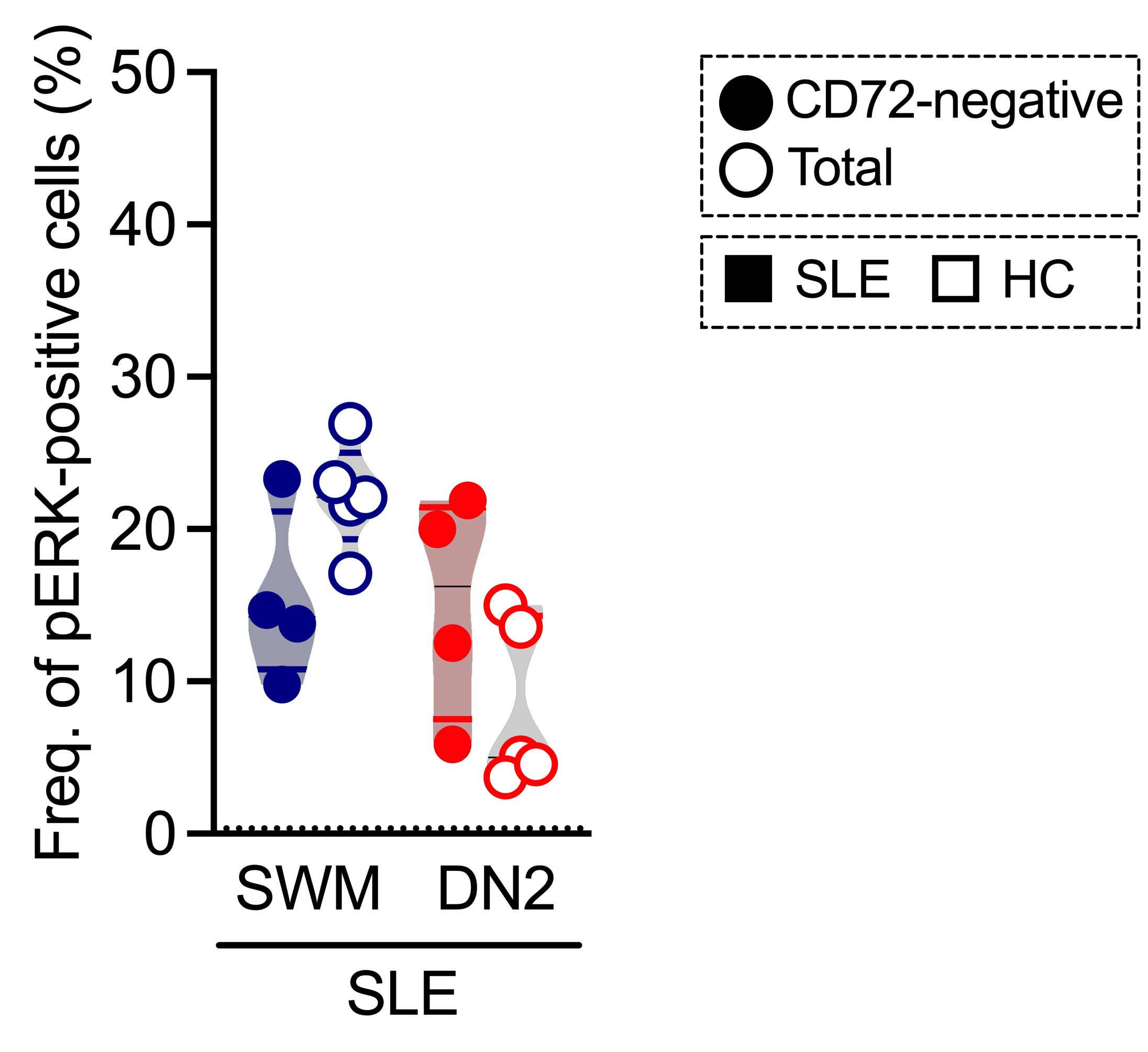


**Supplementary Figure S3** Increased pSYK phosphorylation in lupus CD72-negative SWM and DN2 B cells. Frequencies of pSYK- and pERK-positive cells in CD72-negative SWM and DN2 in SLE patients (n = 4) compared to HC (n = 5). Group comparisons were analyzed by Mann-Whitney U test. Only statistically significant *p* values < 0.05 are presented.
